# Genetic and environmental influences on fatty acid and tocopherol diversity in quinoa germplasm

**DOI:** 10.3389/fpls.2025.1541895

**Published:** 2025-05-15

**Authors:** Leonardo Velasco, Javier Matías, Verónica Cruz, Sara Fondevilla

**Affiliations:** ^1^ Departamento de Mejora Genética Vegetal, Instituto de Agricultura Sostenible – Consejo Superior de Investigaciones Científicas (IAS-CSIC), Córdoba, Spain; ^2^ Centro de Investigaciones Científicas y Tecnológicas de Extremadura (CICYTEX), Instituto de Investigaciones Agrarias Finca La Orden, Badajoz, Spain

**Keywords:** fatty acid profile, genetic diversity, geographic diversity, heritability estimates, oil content, quinoa breeding, tocopherols

## Abstract

**Introduction:**

Quinoa (*Chenopodium quinoa* Willd.) is a food crop highly valued for being nutritious and health-promoting. As quinoa grains contain more oil than cereals, the fatty acid profile is an important nutritional criterion. The tocopherol family, with vitamin E and anti-inflammatory properties, are also relevant compounds for the nutritional quality of quinoa. Currently, information on the genetic diversity of these compounds in quinoa germplasm is limited.

**Methods:**

Fatty acid and tocopherol contents and profiles were analyzed, using gas chromatography and HPLC, respectively, in the seeds of 126 quinoa accessions from diverse origins grown in two locations in Spain in 2021 and 2022, following a completely randomised block design with three replications.

**Results:**

The germplasm exhibited a wide diversity for all the traits analysed, showing contrasting contents and profiles of fatty acids and tocopherols. Considering the data averaged over the four environments, particularly relevant variation was observed for total fatty acid, from 1.0 to 4.4% of grain weight; oleic acid, from 13.4 to 32.3% of total fatty acids; linoleic acid, from 48.0 to 67.4%; linolenic acid, from 1.8 to 8.4%; tocopherol content, from 22.3 to 121.6 mg kg^-1^ grain; α-tocopherol, from 24.6 to 81.4% of the total tocopherols; and γ-tocopherol, from, 18.6 to 75.4%. Broad-sense heritability (H^2^) was found to be high for linolenic acid content (0.86) and the concentrations of α- and γ-tocopherol (0.84). For most of the other traits, H^2^ ranged from 0.65 to 0.77. Principal component analysis successfully separated highland accessions from Bolivia and Peru and lowland accessions from Chile and the USA.

**Discussion:**

The existence of wide genetic diversity for grain oil content, fatty acid profile, and tocopherol content and profile in quinoa germplasm, together with the medium to high heritability of these traits, suggests the feasibility of improving these characters through breeding.

## 1 Introduction

Native to the Andean regions of South America, quinoa (*Chenopodium quinoa* Willd.) is expanding to other cultivation areas, such as the Mediterranean region, because of its outstanding nutritional value (Angeli et al., 2020). Quinoa grains contain from 56.2% to 77.0% carbohydrates, 12.1% to 16.7% proteins of great biological value, 5.5% to 8.5% lipids, and a myriad of minor bioactive compounds (Liu et al., 2024).

The fatty acids are the main components of triacylglycerols and, subsequently, the main contributors to dietary fat. Amongst fatty acids, those with saturated carbon chains are associated with detrimental health effects such as rising total and LDL cholesterol concentrations, whereas unsaturated fatty acids have the opposite impact (Calder, 2015). Quinoa oil is mainly made up of unsaturated fatty acids, which account for 88.5% of the fatty acids, thus being considered a health-promoting food in relation to the fatty acid profile of its oil (Vilcacundo and Hernández-Ledesma, 2017).

Tocopherols and tocotrienols, collectively known as tocochromanols, serve as the primary antioxidants in seeds. They exhibit antioxidant activity both *in vitro* (e.g., in packed food or bottled oils) and *in vivo*, the latter being referred to as vitamin E (Szewczyk et al., 2021). Tocochromanols exist in eight chemical forms, namely α-, β-, γ- and δ-tocopherol and tocotrienol. The α- forms of both tocopherols and tocotrienols demonstrate the highest metabolic effect, i.e., the highest vitamin-E activity (Szewczyk et al., 2021). γ-Tocopherol also showcases unique antioxidant and anti-inflammatory activities, which complement α-tocopherol (Jiang et al., 2022a). In contrast to cereals, which are primarily rich in tocotrienols, quinoa grains are abundant in α- and γ-tocopherol, boasting a higher calculated vitamin-E activity than cereal grains such as wheat and barley (Granda et al., 2018). Thus, Granda et al. (2018) calculated a vitamin E activity of 53,53 α-tocopherol EQ/kg DM for quinoa grains, compared to 11,67 and 16,47 α-tocopherol EQ/kg DM for barley and wheat grains, respectively, showing the superior antioxidant properties of quinoa.

Quinoa germplasm contains large genetic variability for agronomic and grain quality traits (Ng and Wang, 2021; Jiang et al., 2022b; Calderón-González et al., 2023; Craine et al., 2023) and a set of consensual methods has been proposed to evaluate and standardize the phenotyping of this variability (Stanschewski et al., 2021). Furthermore, molecular markers have been used to characterize this genetic diversity, and to identify, through genome-wide association studies and extreme gradient boosting, candidate genes for thousand seed weight, and other agronomic traits, and downy mildew resistance (Christensen et al., 2007; Patiranage et al., 2022; Fondevilla et al., 2024; Sandell et al., 2024; Visintainer et al., 2025).

Genetic diversity for grain quality traits has been determined mainly for protein, fiber, and pigment contents (Bhargava et al., 2007; De Santis et al., 2016). It is important to mention that estimating genetic diversity requires disclosing genetic factors from environmental and management factors (Hatfield and Walthall, 2015), which has not been conducted thus far for the oil content in quinoa grains, its fatty acid composition, and the tocopherol content and composition in the grains, which are the traits evaluated in the present research.

In relation to the oil content in the grains, Rojas et al. (2010) reported a broad range of variation, from 2.1 to 10.9% of grain weight, in a large germplasm collection of 555 Bolivian accessions. However, data reported by Rojas et al. (2010) was based on literature information from several studies (Rojas and Pinto, 2013). Craine et al. (2023) evaluated oil content in a germplasm collection of 360 accessions, including a world core collection, using plants grown in a single environment. Oil content ranged from 0.5 to 6.61% of grain weight.

The fatty acid profile of the oil has been evaluated at a narrower scale. Przybylski et al. (1994) reported that quinoa seeds from a single cultivar contained mainly linoleic acid (56.0% of the total fatty acids), oleic acid (21.1%), and palmitic acid (9.6%) and minor amounts of other fatty acids. Several studies have evaluated variability for fatty acid profile, but in most cases, using a low number of cultivars, from 3 to 21, grown in a single environment or from unreported environments (Miranda et al., 2012; Vidueiros et al., 2015; Duarte et al., 2022; Tang et al., 2016; Pereira et al., 2019).

Similar to the studies on fatty acids, the available reports on tocopherol content and profile in quinoa grains did not consider environmental and management effects. Accordingly, they show total variability, but they did not provide information on how much of this variability was caused by genetic factors. The most comprehensive study evaluated 39 cultivars, most obtained from commercial establishments in Peru and Spain (Pereira et al., 2019). The authors reported an average total tocopherol content of 127.2 mg kg^-1^ seed, made up of γ-tocopherol (88.4% of the total tocopherols), α-tocopherol (10.9%), and δ-tocopherol (0.8%).

The oil content and its fatty acid profile in seed oil are well known to be influenced by environmental factors, mainly the temperature during seed development (Zhang et al., 2015), although other factors, such as the intercepted solar radiation, also have an effect (Izquierdo et al., 2009). In quinoa, Matías et al. (2022) reported that higher temperatures reduced the oil content in quinoa grains and the oleic to linoleic acid ratio in the grain oil. The contents of tocopherols in grains are also exposed to environmental effects since they are involved in plant responses to plant stresses (Faizan et al., 2023).

To the best of our knowledge, there are no studies in the literature that have analysed oil content, fatty acid composition, and tocopherol content and profile of quinoa seeds in a wide collection of genetically diverse accessions in different environments. These kinds of studies are needed to accurately estimate environmental and genetic effects on these traits, which are essential for predicting responses to selection. Accordingly, the objective of the present research was to analyse fatty acid and tocopherol contents and profiles in a world collection of quinoa germplasm grown in four different environments and to evaluate the genetic and environmental effects and the heritability of the analysed traits.

## 2 Materials and methods

### 2.1 Plant material and experimental design

Around 300 accessions were initially obtained from the USDA North Central Regional Plant Introduction Station of the US National Plant Germplasm System (USA) and IPK Gatersleben (Germany) genebanks. To increase the homogeneity of each accession before carrying out the field experiments, one plant per accession was selected and selfed at least twice. During this process, those plants that did not produce seeds under the local conditions were discarded (Calderón-González et al., 2023). Finally, a collection of 216 accessions, including the cultivars Atlas, F16, Vikinga, and Duquesa, that were provided by the company Algosur S.A., was selected for the field experiments. The accessions were grown in two locations in Spain in 2021 and 2022: Córdoba (longitude: 37°51’48”N; latitude: 4°47’25”W; altitude: 91 m) and Guadajira (longitude: 38°51′07″N; latitude: 6°40′49″O; altitude: 222 m). The soil in Córdoba is sandy loam and classified as Xerofluvent, whereas the soil in Guadajira is clay loam and classified as Xerorthent (Soil Survey Staff, 2006). Environmental conditions during the field experiments were different between the two locations (**Supplementary Table 1**). Since temperatures in February were lower in Guadajira than in Córdoba, sowing dates were later in Guadajira (25^th^ February in 2021 and 3^rd^ March in 2022) than in Córdoba (3^rd^ February in 2021 and 15^th^ February in 2022). Accessions were sown according to a completely randomised block design with three replications and grown under rainfed conditions, using similar agronomic practices in both locations. In each block, the accessions were grown in 1-m row (10 plants per row) with 0.7-m separation between rows. Basal fertilization of 400 kg/ha of 8:15:15 N:P:K fertilizer plus 87 kg of urea (N, 46%)/ha was applied one week before sowing. A top dressing of 130 kg urea/ha was applied during flowering. Several accessions did not adapt properly to the local conditions and did not flower or produce enough seeds for grain quality analyses. So, finally, only the 126 accessions that produced enough seeds for grain quality analyses were included in the study. The selection was based on the criteria of including accessions that produced enough seeds in at least two environments and four replications, although most of the accessions produced enough seeds in the four environments. The analysis included an average of 3.8 environments and 9.2 replications per accession. The accessions included in the analysis are listed in **Supplementary Table 2**.

### 2.2 Fatty acid analyses

Sixteen grains were weighed and placed in a 2-mL glass vial, where 0.55 mL of heptane, 0.75 mL of methylation reagent, and 0.20 mL of an internal standard solution of 6 mM heptadecanoic acid in heptane were added. The methylation reagent was prepared with methanol (66% by vol.), toluene (28% by vol), 2,2-dimethoxypropane (4% by vol.), and sulfuric acid (2% by vol.) The vials were maintained for two hours in a water bath at 80 °C (Garcés and Mancha, 1993). One µL of the upper layer, containing the fatty acid methyl esters, was injected into a Perkin Elmer Clarus 600 gas chromatograph (Perkin Elmer Inc, Waltham, MA, USA) equipped with a split/splitless injector, a flame ionization detector (FID) and a capillary column BPX70–30 m length x 0.25 mm internal diameter x 0.25 µm film thickness (SGE Analytical Science Pty Ltd, Ringwood, Australia). The carrier gas was hydrogen, at a constant flow of 0.8 mL min^-1^. The injector was set at 280 °C, with a split flow of 100 mL min^-1^. The FID was set at 300 °C. The initial oven temperature was 180 °C maintained for two minutes, followed by a rate increase of 10 °C min-1 up to 230 °C, maintained for two minutes. Fatty acids were identified based on their retention times compared to control samples of low- and high-erucic acid *Brassica carinata* A. Braun accessions with known fatty acid profiles (Velasco et al., 2004). The concentration of each fatty acid was computed as the percentage of the area of the fatty acid in relation to the sum of the areas of all fatty acids except the internal standard.

Conventional methods for the analysis of grain oil content, such as Soxhlet extraction, require a large amount of grains, e.g., 10 g in the study of Etaati et al. (2023). In the present study, based on a high number of accessions from different origins, some of the accessions did not produce enough grains for such analyses. Alternatively, this trait was computed following the method proposed by Kumar and Fujimoto (1977) based on the estimation of oil content as the total content of fatty acids in the grains. The estimation is possible thanks to using a known amount of heptadecanoic acid as an internal standard. The main advantage of this method is that it allows the analysis of small samples (sixteen grains were used in the present study). Conversely, the main limitation of the method is that it underestimates the grain oil content (Kumar and Fujimoto, 1977). Such underestimation is of low magnitude, considering that fatty acids represent around 85% of the quinoa oil (Wood et al., 1993). Also, a similar underestimation is expected for all the samples, which allows a comparative analysis within the germplasm collection. Based on the previous premises, the total fatty acid content in the grains was used to estimate the genetic variability for grain oil content, considering the impracticability of measuring oil content accurately in this experiment. For simplicity and ease of understanding, we will use grain oil content throughout the manuscript, even though we will refer to grain fatty acid content, expressed as the percentage of fatty acids in the grain on a dry weight basis. Moisture content was calculated from the fresh and dry weight of at least 300 mg of seeds from the same set used for the analyses. The seeds were dried at 100°C for 48 h.

### 2.3 Tocopherol analyses

Tocopherol analyses were based on the method proposed by Goffman et al. (1999), with some modifications. Sixteen seeds were placed in a 10-mL polypropylene tube and finely crushed with a stainless-steel rod. After weighing the resulting flour, 3 mL isooctane and 1 mL of a solution 12 mM of Rac-5,7-dimethyltocol (Matreya LLC, Pleasant Gap, PA, USA) in isooctane, used as internal standard, were added. The tube was vortexed, maintained at room temperature in the dark overnight, and vortexed again before filtering the extract using nylon syringe filters with 0.45 µm of pore size. The filtered extract was analyzed by high-performance liquid chromatography (HPLC) using an eluent composed of isooctane (94% by vol.) and tert-butylmethylether (6% by volume) at a constant flow of 0.8 ml/min. Tocopherols were separated using a LiChrospher 100 diol column (0.25 m length, 3 mm internal diameter; Merck KGaA, Darmstadt, Germany) with 5-μm spherical particles, connected to a silica guard column (LiChrospher Si 60, 5 mm length, 4 mm internal diameter; Merck KgaA, Darmstadt, Germany). Tocopherol standards (Calbiochem Tocopherol Set, Cat. No. 613424, Merck KgaA, Darmstadt, Germany) were used to identify the individual tocopherols based on their retention times. Total tocopherol content was calculated as the sum of the individual tocopherols and expressed as mg per kg of grain on a dry weight basis. Since high-temperature treatment may alter tocopherol content in seeds (Potočnik et al., 2018), analyses were conducted on undried seeds, then the results were corrected based on the moisture content of the seeds. The concentration of individual tocopherols was reported as a percentage of the total tocopherols.

### 2.4 Statistical analyses

An analysis of variance was conducted for each trait using the genotype, the location, and the year as fixed factors. Broad-sense heritability (H^2^) was computed as the proportion of the genotypic variance in relation to the total variance (Steel and Torrie, 1980). Pearson’s correlation coefficients between traits were calculated using the averaged values of each accession across the four environments. Principal component analysis (PCA) with varimax rotation was performed on all the quantitative variables using the average for every variable across the four environments. To study if the area of origin of the accessions influenced the grain quality traits, ANOVA was conducted using the country of origin as the dependent variable in a subset of 115 accessions for which the country of origin was known. The mean values per country were compared using Tukey’s *post hoc* test for multiple comparisons. PCA with varimax rotation was also repeated, including the subset of 115 accessions. All the analyses were performed using IBM SPSS Statistics v 22 (IBM Corp., Armonk, NY, USA).

## 3 Results

Grain oil content in the germplasm collection averaged 2.8%, ranging from 1.0 to 4.4% at the individual accession level (**Table 1**). The fatty acid profile of the oil was mainly made up of 59.5% linoleic acid (48.0 to 67.4%), 19.8% oleic acid (13.4 to 32.3%) and 10% palmitic acid (8.0 to 12.5%). Linolenic acid content averaged 5.3% and exhibited a broad range of variation, from 1.8 to 8.4%, whereas erucic acid content averaged 1.6% and ranged from 0.8 to 2.4%.

**Table 1:** Total mean and standard error (SE), and lowest and highest mean accession values for grain oil content (measured as % dry grain weight), concentration of palmitic, stearic, oleic, linoleic, linolenic, eicosenoic and erucic acid in the grain oil (measured as % of total fatty acids), total tocopherol content (mg kg^-1^), and concentrations of α- and γ-tocopherol (measured as % of tocopherols) in a collection of 126 quinoa accessions grown in Córdoba and Guadajira (Spain) in 2021 and 2022.

Trait	Mean	SE	Lowest	Highest
Grain oil content	2.8	0.3	1.0	4.4
Palmitic acid	10.1	0.9	8.0	12.5
Stearic acid	0.7	0.1	0.5	1.3
Oleic acid	19.8	1.8	13.4	32.3
Linoleic acid	59.5	5.3	48.0	67.4
Linolenic acid	5.3	0.5	1.8	8.4
Eicosenoic acid	1.3	0.1	0.8	2.0
Erucic acid	1.6	0.1	0.8	2.4
Tocopherol content	58.2	5.2	22.3	121.6
α-Tocopherol	53.3	4.8	24.6	81.4
γ-Tocopherol	46.6	4.2	18.6	75.4

Larger variability was observed for tocopherol content and profile in the quinoa germplasm studied. Total tocopherol content averaged 58.2 mg kg^-1^ and exhibited a broad variation from 22.3 to 121.6 mg kg^-1^. Interestingly, contrasting tocopherol profiles were observed, with either high levels of α or γ-tocopherol. Thus, the proportion of α-tocopherol ranged from 24.6 to 81.4% of the total tocopherols, whereas γ-tocopherol ranged from 18.6 to 75.4%. The average grain oil content, fatty acid composition, and tocopherol content and composition of individual accessions are provided in **Supplementary Table 2**.

The analysis of variance revealed that the genotype and the location had a significant effect (*p*<0.01) on all the traits. The year was also significant, except for the concentrations of eicosenoic and erucic acid. The interactions were also highly significant in most cases, with the exceptions that can be observed in **Table 2**. Most relevant was the reduced statistical significance (*p*<0.05) for the genotype x location (G x L) interaction for gran oil content and the genotype x year (G x Y) interaction for total tocopherol content, and the non-significant location x year (L x Y) interaction for the concentrations of linoleic and linolenic acid (**Table 2**).

**Table 2:** Analysis of variance (sums of squares) and estimates of broad-sense heritability (H^2^) for grain oil content (measured as % dry grain weight), concentration of major fatty acids in the oil (measured as % of total fatty acids), total tocopherol content (mg kg^-1^ grain weight), and concentrations of α- and γ-tocopherol (measured as % tocopherols) in a collection of 126 quinoa accessions grown in Córdoba and Guadajira (Spain) in 2021 and 2022.

Trait	Genotype (G)	Location (L)	Year (Y)	G × L	G x Y	L x Y	G x L x Y	Error	H^2^
Grain oil content	784.2^**^	8.3^**^	16.8^**^	49.5^*^	53.2^**^	52.1^**^	42.8^**^	182.5	0.76
Palmitic acid	715.3^**^	10.2^**^	4.2^**^	133.7^**^	124.3^**^	89.8^**^	84.5^**^	348.5	0.57
Stearic acid	16.5^**^	0.6^**^	0.6^**^	10.3^**^	10.3^**^	0.9^**^	5.6	32.9	0.20
Oleic acid	10021.2^**^	35.2^**^	17.7^**^	727.2^**^	850.5^**^	18.7^**^	540.2^**^	1682.2	0.76
Linoleic acid	7862.3^**^	50.0^**^	24.7^**^	985.0^**^	932.8^**^	0.8	535.9^**^	1896.0	0.68
Linolenic acid	1879.2^**^	30.0^**^	20.5^**^	86.5^**^	71.6^**^	1.2	47.2^**^	210.6	0.86
Eicosenoic acid	37.5^**^	0.9^**^	0.0	2.8^**^	2.8^**^	3.3^**^	2.7^**^	9.7	0.77
Erucic acid	62.5^**^	22.3^**^	0.1	7.8^**^	7.9^**^	4.0^**^	6.8^**^	21.5	0.65
Tocopherol content	263100.7^**^	21037.8^**^	51419.4^**^	76941.4^**^	50980.0^*^	2732.1^**^	34324.8	212449.5	0.47
α-Tocopherol	123748.6^**^	6813.2^**^	1298.7^**^	6915.6^**^	4902.6^**^	368.7^**^	3394.3^**^	13135.7	0.84
γ-Tocopherol	123473.8^**^	6875.9^**^	1381.7^**^	6877.7^**^	4892.9^**^	376.1^**^	3404.5^**^	13243.3	0.84

*Significant at *p*<0.05; **Significant at *p*<0.01.

Broad-sense heritability (H^2^) was particularly low for the concentration of stearic acid (0.20), total tocopherol content (0.47), and palmitic acid content (0.57). Higher heritability values were observed for erucic acid concentration (0.65), linoleic acid concentration (0.68), grain coil content and oleic acid concentration (0.76), eicosenoic acid concentration (0.77), α- and γ-tocopherol concentrations (0.84), and linolenic acid concentration (0.86; **Table 2**).

**Figure 1:**
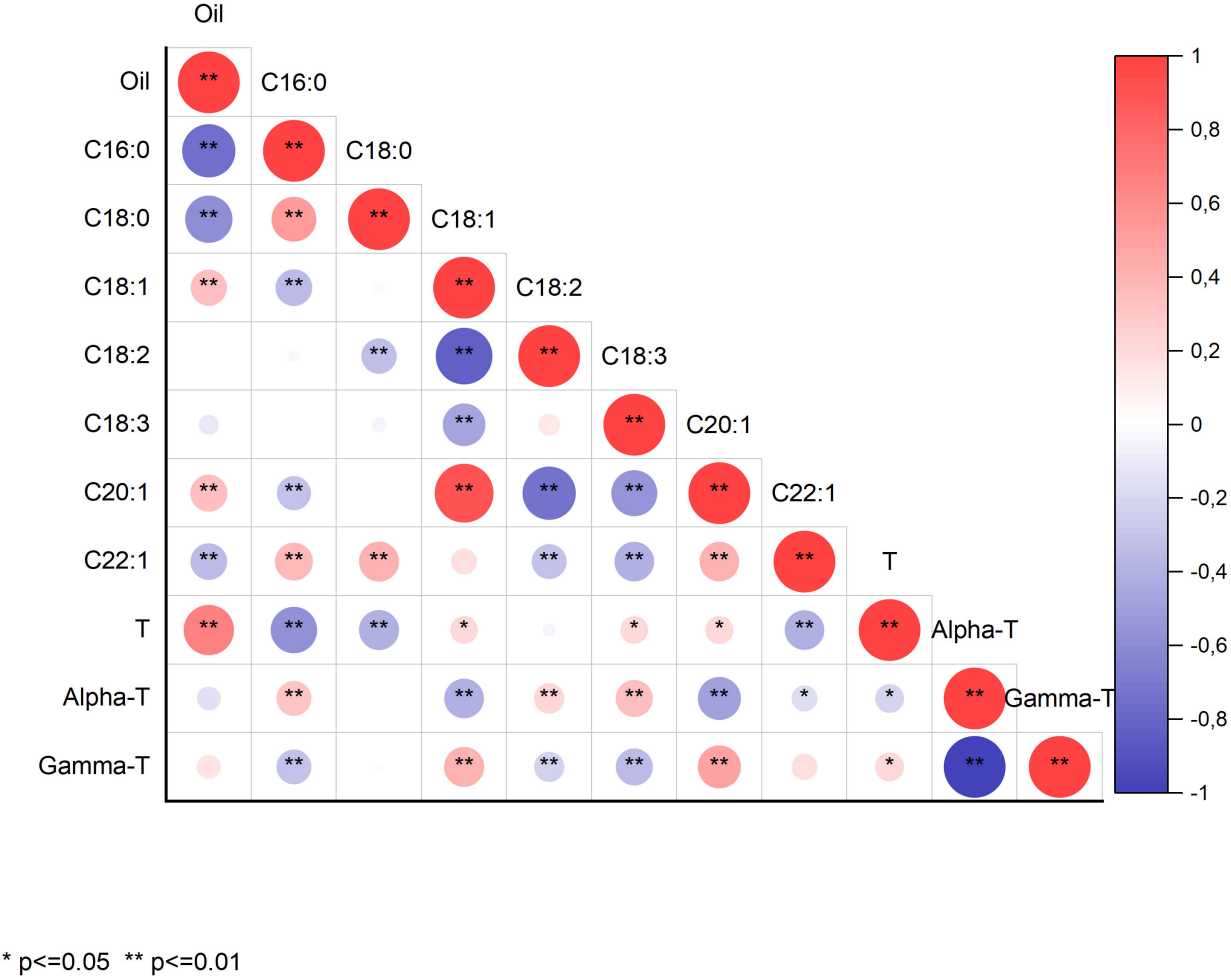
Correlation coefficients between grain oil content (Oil), concentrations of the fatty acids palmitic (16:0), stearic (18:0), oleic (18:1), linoleic (18:2), linolenic (18:3), eicosenoic (20:1), and erucic acid (22:1), total tocopherol (T) content, and concentrations of α- and γ-tocopherol (α-T and γ-T) in a collection of 126 quinoa accessions grown in Córdoba and Guadajira (Spain) in 2021 and 2022. *Significant at *p*<0.05; **Significant at *p*<0.01.

Concerning highly significant correlation coefficients (*p*<0.01), we observed that oil content was negatively correlated with the concentration of the saturated fatty acids palmitic (r=-0.74) and stearic acid (r=-0.59), positively correlated with the monounsaturated fatty acids oleic (r=0.35) and eicosenoic acid (r=0.36), and negatively correlated with the monounsaturated erucic acid (r=-0.35; **Figure 1**). Oil content was also positively correlated with total tocopherol content (r=0.67) but not with the proportion of individual tocopherols. The correlation of total tocopherol content with the concentration of individual fatty acids followed the same trend as the correlation between oil content and fatty acids, probably due to the mentioned strong positive correlation between oil and tocopherol contents (**Figure 1**).

Palmitic acid was positively correlated with stearic acid (r=0.53) and erucic acid (r=0.38) and negatively correlated with oleic (r=-0.35) and eicosenoic acid (r=-0.30). Similar correlations were observed for stearic acid (**Figure 1**). Oleic acid showed a strong negative correlation with linoleic acid (r=-0.84) and a positive correlation with eicosenoic acid (r=0.90). It was also negatively correlated with linolenic acid (r=-0.48). Interestingly, no significant correlation was observed between linoleic and linolenic acids, whereas they both showed negative correlations with eicosenoic and erucic acid concentrations (**Figure 1**). Finally, eicosenoic and erucic acid concentrations were positively correlated (r=0.41).

Total tocopherol content showed a significant (*p*<0.05) negative correlation with the proportion of α-tocopherol (r=-0.23) and a concomitant positive correlation with the proportion of γ-tocopherol (r=0.23). Since the individual tocopherols are expressed as a percentage of the total tocopherols and only α- and γ-tocopherol were detected, they showed a complete negative correlation (r=-1.0).

**Figure 2:**
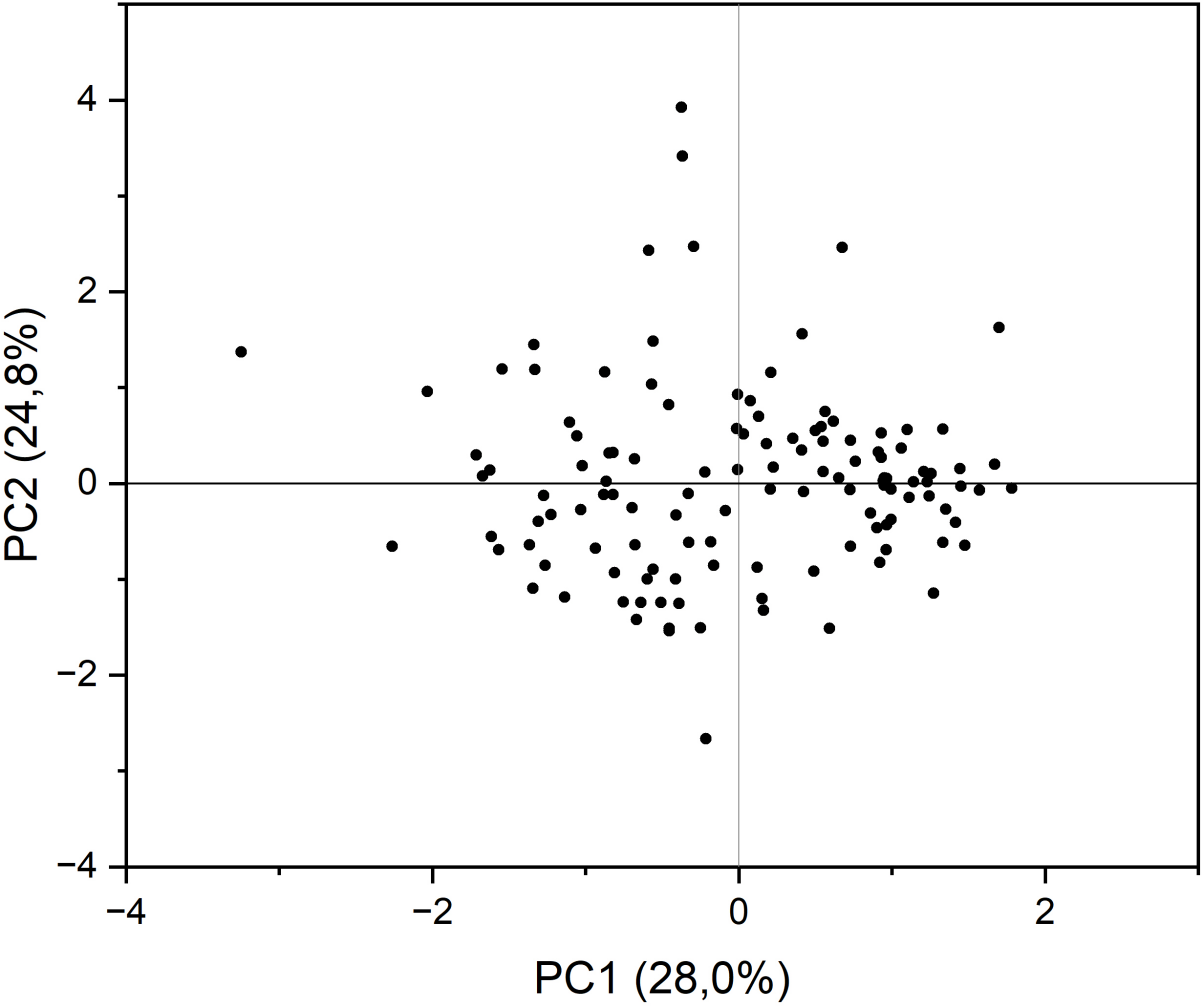
Biplot of principal component (PC) 1 *vs*. PC 2 from principal component analysis conducted on the grain quality components analysed in a collection of 126 quinoa accessions grown in Córdoba and Guadajira (Spain) in 2021 and 2022.

Principal components analysis (PCA) with varimax rotation was conducted to study how the accessions were classified based on the grain quality traits evaluated and which traits had a major weight in such a classification. The four principal components explained 86.3% of the total variance together, with individual contributions from 28.0% of PC1 to 13.8% of PC4 (**Table 3**). The major loadings in the components corresponded to oil content, palmitic acid concentration, stearic acid concentration, tocopherol content, and erucic acid concentration (PC1); linoleic, oleic, and eicosenoic acid concentration (PC2); concentration of α- and γ-tocopherol (PC3); and linolenic acid concentration (PC4; **Table 3**). **Figure 2** shows the biplot of PC1 versus PC2.

**Table 3:** Component loadings for principal components (PC) 1 to 4 in the analysis of oil content, concentration of the fatty acids palmitic, stearic, oleic, linoleic, linolenic, eicosenoic, and erucic acid in the oil, total tocopherol content, and concentrations of α- and γ-tocopherol in a collection of 126 quinoa accessions grown in Córdoba and Guadajira (Spain) in 2021 and 2022.

Trait	PC1 (28.0%)	PC2 (24.8%)	PC3 (19.7%)	PC4 (13.8%)
Oil content	0.90	0.19	0.02	-0.07
Palmitic acid	-0.83	-0.13	-0.25	-0.04
Stearic acid	-0.80	0.25	0.06	0.09
Oleic acid	0.21	0.90	0.20	-0.24
Linoleic acid	0.19	-0.96	-0.08	0.07
Linolenic acid	-0.02	-0.20	-0.21	0.89
Eicosenoic acid	0.19	0.82	0.28	-0.41
Erucic acid	-0.55	0.27	0.12	-0.54
Tocopherol content	0.71	0.19	0.21	0.40
α-Tocopherol	-0.09	-0.18	-0.97	0.13
γ-Tocopherol	0.09	0.18	0.97	-0.14

The percentage of the variance explained by each PC is given in brackets.

**Figure 3:**
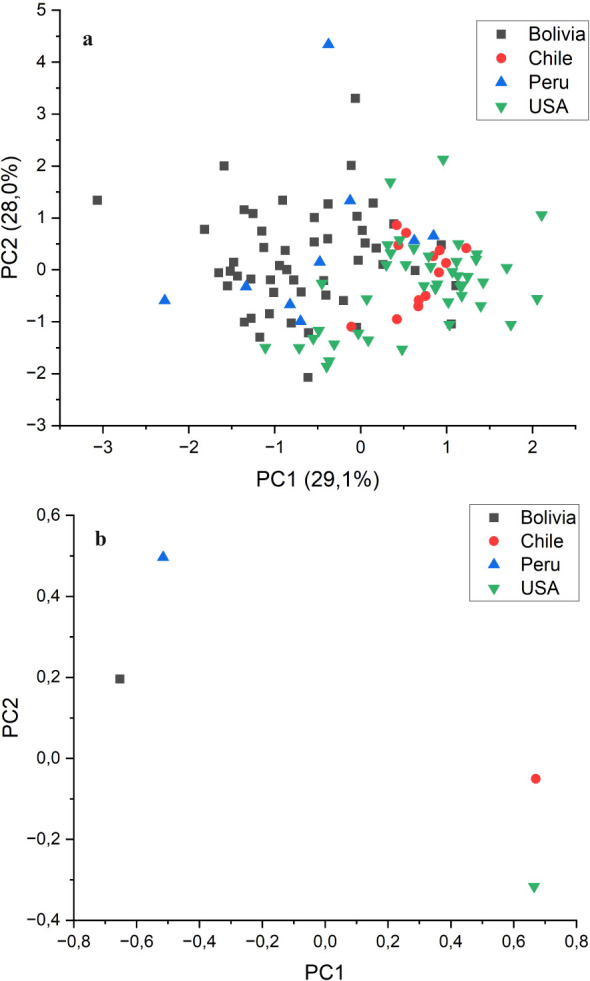
Biplot of principal component (PC) 1 *vs*. PC 2 from principal component analysis conducted on the grain quality components analysed in 115 quinoa accessions, grown in Córdoba and Guadajira (Spain) in 2021 and 2022, grouped by country of origin. **(a)** Individual accessions; **(b)** averages by country.

An ANOVA analysis using the country of origin of the accessions as the dependent variable was conducted to test whether the precedence of the accessions had an effect of the grain quality traits evaluated in this study. The results showed that the country of origin had a significant effect (*p*<0.01) on most of the traits, with the exceptions of α- and γ-tocopherol concentrations, for which it was also significant but at *p*<0.05, and oleic acid and eicosenoic acid concentrations, for which the origin was not significant. **Table 4** shows the average values per country. On average, the accessions from Chile and the USA had higher oil content, with lower palmitic and erucic acid concentrations than those from Bolivia and Peru. Significantly, higher tocopherol content was observed in the accessions from the USA. In contrast, the accessions from Chile and Peru showed contrasting tocopherol profiles, with those from Chile having a predominance of α-tocopherol (60.9%) and those from Peru showing a predominance of γ-tocopherol (53.1%) (**Table 4**). PCA indicated that populations from Chile and the USA, as well as populations from Bolivia and Peru, were consistently grouped, delineating distinct clusters (**Figure 3a**). This was more clearly seen when the average values for each country were plotted instead of the values of the individual accessions (**Figure 3b**).

**Table 4:** Significance level of ANOVA conducted using the country of origin as the dependent variable and average values of grain oil content, concentration of the fatty acids palmitic, stearic, oleic, linoleic, linolenic, eicosenoic, and erucic acid in the oil, total tocopherol content, and concentrations of α- and γ-tocopherol in quinoa accessions from Bolivia (n=50), Chile (n=13), Peru (n=9), and USA (n=43), grown in Córdoba and Guadajira (Spain) in 2021 and 2022.

Trait	Significance	Bolivia^1^	Peru	Chile	USA
Oil content (% grain)	<0.01	2.2^a^	2.3^a^	3.7^b^	3.3^b^
Palmitic acid (% fatty acids)	<0.01	10.5^b^	10.5^b^	9.7^a^	9.6^a^
Stearic acid (% fatty acids)	<0.01	0.8^b^	0.7^ab^	0.6^a^	0.6^a^
Oleic acid (% fatty acids)	>0.05	20.0	21.6	19.8	19.5
Linoleic acid (% fatty acids)	<0.01	58.8^ab^	57.7^a^	60.4^b^	60.0^b^
Linolenic acid (% fatty acids)	<0.01	5.0^ab^	4.4^a^	5.1^ab^	6.0^b^
Eicosenoic acid (% fatty acids)	>0.05	1.3	1.3	1.2	1.2
Erucic acid (% fatty acids)	<0.01	1.8^b^	1.7^b^	1.5^a^	1.4^a^
Tocopherol content (mg kg^-1^ grain)	<0.01	51.9^a^	53.2^a^	61.5^ab^	66.8^b^
α-Tocopherol (%tocopherols)	<0.05	52.3^ab^	46.9^a^	60.9^b^	53.6^ab^
γ-Tocopherol (%tocopherols)	<0.05	47.6^ab^	53.1^b^	39.1^a^	46.3^ab^

^1^Within a row, means followed by the same letter are not significantly different at the significance level indicated based on Tukey’s *post-hoc* analysis.

## 4 Discussion

Climate change scenarios forecast a pronounced decline in precipitation, with increased temperatures and evaporative demand (Gómez-Gómez et al., 2022). Consequently, climate-change-resilient crops are expected to play a key role in the provision of sustainable food. Quinoa is considered a climate change-resilient crop, due to its intrinsic tolerance to drought, and is gaining worldwide attention due to its outstanding nutritional value (Fondevilla et al., 2024). The results of this study, based on the evaluation of 126 accessions under four environments, revealed that quinoa germplasm contains significant genetic variation for grain quality traits such as oil content, fatty acid profile, and tocopherol content and profile. Through our study, a set of quinoa accessions showing outstanding values for these nutritional traits, under rainfed conditions, was identified, which lays the foundations for the development of new quinoa varieties capable of producing high-quality food under a climate-change scenario. The use of an extensive germplasm collection and the experimental conditions based on four environments allowed genetic and environmental effects to be disclosed from the observed phenotypic variability. This is important because environmental effects greatly influence grain quality traits in quinoa (Curti et al., 2020; Granado-Rodríguez et al., 2021; Matías et al., 2022). Most previous studies on quinoa diversity related to grain quality traits have involved a limited number of accessions and have not distinctly separated genetic from environmental effects on the evaluated traits (e.g. Wood et al., 1993; Miranda et al., 2012; Tang et al., 2015; Vidueiros et al, 2015; Tang et al., 2016; Granda et al., 2018; Pereira et al., 2019; Duarte et al., 2022). Other studies were based on germplasm evaluated under several environments, although focused on a lower number of accessions. Etaati et al. (2023) evaluated ten accessions of quinoa in four environments for oil content, fatty acid profile, and other nutritional traits. Präger et al. (2018) evaluated four accessions for oil content and fatty acid and amino acid profiles under two environments. In both studies, the ranges of variation of the studied traits were narrower than those identified in the present study. For example, linoleic acid content ranged from 58.8% to 62.6% in the study of Etaati et al. (2023), and from 57.4% to 61.0% in the study of Präger et al. (2018), compared to 48.0% to 67.4% in the present study. Similarly, linolenic acid content ranged from 4.9% to 6.2% in the study of Etaati et al. (2023), and 3.3% to 5.0% in the study of Präger et al. (2018), compared to 1.8% to 8.4% in the present study. The influence of the environmental conditions on the fatty acid profile of the oil in quinoa grains has also been analyzed in a previous study, concluding that high temperatures promote a reduction in the oil content and the oleic to the linoleic acid ratio (Matías et al., 2022). Our research focused on evaluating the relative weight of genetic and environmental factors, but the study of the nature of environmental effects was out of the scope of the work.

Total oil content widely differed between the two studies conducted in more than one environment, from 3.0% to 3.7% in the study of Etaati et al. (2023) and 5.5% to 7.5% in Präger et al. (2018). Such a difference may be caused by using different analytical methodologies, contrasting cultivars, or strong environmental effects. Our results, based on the analysis of the total fatty acid content as described in the methodology, align with the results of Etaati et al. (2023), with a broader range of variation between 1.0 and 4.4%.

Several studies have reported tocopherol content and profile in quinoa germplasm, but to the best of our knowledge, they have all been conducted without considering the strong influence of the environment on these compounds (Ghosh et al., 2021). The most comprehensive study included 39 accessions for which the analyzed grains were obtained from different origins, thus reflecting unknown environmental effects. In any case, the authors did not report information on individual accessions but average values for grain color groups. Thus, the average total tocopherol content ranged from 971 μg 100 g^-1^ (=97.1 mg kg^-1^) in accessions with white grains to 1764 μg 100 g^-1^ (=176.4 mg kg^-1^) in cultivars with black grains. Minor differences were observed among groups for the relative proportion of the individual tocopherols, with gamma-tocopherol being clearly predominant in the three groups, ranging from 86% of the total tocopherols in white seeds to 92% of the total tocopherols in black seeds. As the authors concluded, differences in tocopherols among the studied groups were not necessarily inherent to grain color, but they might have been caused by the storage time and conditions and the agronomic and environmental conditions under which the accessions were grown (Pereira et al., 2019). In the present study, the accessions exhibited large genetic diversity not only for total tocopherol content (22.3 to 121.6 mg kg^-1^) but also for the proportion of the individual tocopherols, from 24.6 to 81.4% of the total tocopherols for α-tocopherol, and from 18.6 to 75.4% of the total tocopherols for γ-tocopherol. Marked differences between the highest total tocopherol content reported by Pereira et al. (2019) and the highest content found in the collection evaluated in the present research may probably respond to differences in the analytical conditions or to environmental effects since the samples used for the present study were analyzed shortly after harvest, which discards possible losses of tocopherols caused by storage.

The tocopherols are a family of four compounds named α-, β-, γ-, and δ-tocopherol that differ in their *in vivo* and *in vitro* effects. α-tocopherol has the highest *in vivo* activity as vitamin E antioxidant, whereas γ-tocopherol is mainly associated with *in vivo* anti-inflammatory activity and *in vitro* thermoxidative effect (Szewczyk et al., 2021). Since quinoa is a grain used primarily for human food, its vitamin E value is particularly relevant. In this sense, the existence in the germplasm of accessions with a very high proportion of α-tocopherol up to 81.4% of the total tocopherols is of great relevance to breeding for cultivars with increased vitamin E value, given that other previous studies on the analysis of tocopherols in quinoa germplasm found a predominance of γ-tocopherol (Tang et al., 2015, 2016; Granda et al., 2018; Pachari Vera et al., 2019).

Breeding for grain quality traits requires previous knowledge of the relative weight of genetic factors on the expression of the trait, i.e., its heritability. The present study revealed, for the first time, heritability values for oil content, fatty acid profile, and the tocopherol content and profile. Heritability values were exceptionally high for linolenic acid content (H^2^ = 0.86) and the proportions of α- and γ-tocopherol (H^2^ = 0.84), showing that, although the environment has some effect on these traits, there is high genetic variability available for breeding for these compounds. Linolenic acid is an essential omega-3 fatty acid that is associated with a reduced incidence of cardiovascular diseases and type 2 diabetes. There is also promising evidence of its role in counteracting cognitive impairment (Sala-Vila et al., 2022). The health implications of tocopherols have been discussed above. High heritability values (>0.75) were also observed for other fatty acids, such as palmitic and oleic, with an impact on human health, negative in the case of palmitic acid and positive in the case of oleic acid (Sellem et al., 2022). Correlation analysis showed that the proportions of both fatty acids show a significant negative correlation (r=-0.35), pointing to the feasibility of simultaneously increasing oleic acid content and reducing palmitic acid. Conversely, the negative correlation between oleic acid and linolenic acid (r=-0.48) suggests that it might be difficult to increase simultaneously the levels of both fatty acids with positive health effects. Oil content was positively correlated with total tocopherol content but not with the proportion of individual tocopherols, suggesting that it will be possible to select simultaneously for increased oil and tocopherol contents, and specific tocopherol profiles such as high proportion of α-tocopherol, which exerts higher *in vivo* vitamin-E activity (Szewczyk et al., 2021). The existence of correlations between the concentrations of individual fatty acids and tocopherols also requires attention. Thus, the proportion of α-tocopherol was positively correlated with the concentration of palmitic acid (r=0.35) and linolenic acid (r=0.36), and negatively correlated with the concentration of oleic acid (r=-0.41), which might hinder increasing oleic acid and reducing palmitic acid while increasing the levels of α-tocopherol. The variability described in this study will facilitate further studies to identify molecular markers linked to fatty acid and tocopherol traits, which will in turn ease the combination of interesting quality profiles in a single quinoa variety.

It is noteworthy that PCA, using the grain quality traits studied in this research as primary variables, clearly separated the accessions into two groups based on their areas of origin: accessions from Bolivia and Peru on one side and accessions from Chile and the USA on the other. The results are in concordance with the two germplasm pools reported in quinoa, highland (from Bolivia and Peru) and lowland (Chile and USA) (Anuradha et al., 2023). In a previous study, the analysis of SNPs and SilicoDarT markers derived from the sequencing of the same quinoa collection used in this study clearly differentiated, genetically, highland from lowland accessions (Fondevilla et al., 2024). Furthermore, the mentioned study also showed a correlation between the response of the quinoa accessions to the pathogen *P. variabilis* and the geographical group (highland or lowland). Similarly, another PCA study, based on single nucleotide polymorphisms (SNPs) of 310 quinoa accessions, also differentiated most accessions from highland locations from those from lowland locations (Patiranage et al., 2022). Our study, based on grain quality traits, clearly supports the differentiation into highland and lowland quinoa previously reported in these other studies. Adaptation to altitude has been reported to be associated with the fatty acid profile in other crops. For example, higher altitudes resulted in higher oleic acid content and lower palmitoleic and linoleic acid content in avocados (Carvalho et al., 2015). Altitude was negatively correlated with the proportions of α- and δ-tocopherol and positively correlated with the proportion of γ-tocopherol in soybean accessions (Ghosh et al., 2021). Although direct comparison with other crops is not possible, particularly in the case of avocado, in which the oil is mainly in the fruit flesh, our results provide additional support to previous observations in which adaptation to high altitudes has been associated with modifications in the fatty acid and tocopherol profiles of the seed or fruit. It is important to mention that our study does not reflect the influence of altitude on fatty acids and tocopherols, observed in many crops (e.g. Beyhan et al., 2011), since all the accessions have been cultivated in environments with similar altitudes in Spain, but the possible influence of adaptation to high altitudes on the genetic systems controlling biosynthetic pathways of fatty acids and tocopherols.

## 5 Conclusions

This research significantly enhances our understanding of quinoa genetic variability in grain oil content, fatty acid profile, and tocopherol content and profile. Through the screening of a wide quinoa germplasm collection, we have corroborated the large variability present in this crop for these traits, identifying wider ranges than those that had been described so far. In addition, the evaluation of the collection in four environments allowed us to elucidate the influence of genotype, environmental factors, and areas of origin on these traits, which had not been done before. Furthermore, we have calculated the heritability of these traits, which is a relevant parameter for breeding. Heritability of most of the traits was shown to be medium to high, which suggests the feasibility of conducting breeding research focused on specific nutritional objectives. Since quinoa is marketed as a healthy food, breeding for certain grain quality traits such as increasing oleic and linolenic acid content, tocopherol content, and the proportion of α-tocopherol, the most active form of vitamin E, is highly relevant. Moreover, the correlation analysis provides useful information to predict the feasibility of simultaneously selecting several of the traits analyzed in this study.

## Data Availability

The original contributions presented in the study are included in the article/**Supplementary Material**. Further inquiries can be directed to the corresponding author.
