# Supplementary material for: Genetic and environmental influences on fatty acid and tocopherol diversity in quinoa germplasm
Source: Front Plant Sci. 2025 May 15;16:1541895. doi: 10.3389/fpls.2025.1541895 (PMC12124127; doi:10.3389/fpls.2025.1541895)
Supplement: Supplementary file 1 [file Table1.docx]

**Supplementary Table 1**: Average mean temperature (T_m_), average minimum temperature (T_min_) and average maximun temperature (T_max_) during seed feeling, and total precipitation (mm) during seed feeling (P_SF_) and during the whole crop cycle (P_CC_), in the field experiments carried out in Córdoba and Guadajira in 2021 and 2022

| **Location** | **Year** | **T_m_** | **T_min_** | **T_max_** | **P_SF_** | **P_CC_** |
| --- | --- | --- | --- | --- | --- | --- |
| Córdoba | 2021 | 24.34 | 16.52 | 31.66 | 93.00 | 146.20 |
| Córdoba | 2022 | 23.28 | 14.66 | 31.93 | 56.20 | 207.00 |
| Guadajira | 2021 | 21.72 | 13.28 | 29.85 | 37.16 | 106.87 |
| Guadajira | 2022 | 26.77 | 17.41 | 35.75 | 0 | 113.11 |
